# Cooperative roles of introns 1 and 2 of tobacco resistance gene *N* in enhanced *N* transcript expression and antiviral defense responses

**DOI:** 10.1038/s41598-021-94713-4

**Published:** 2021-07-29

**Authors:** Chihiro Ikeda, Kazuo Taku, Tsumugi Miyazaki, Rikako Shirai, Richard S. Nelson, Hiroshi Nyunoya, Yasuhiko Matsushita, Nobumitsu Sasaki

**Affiliations:** 1 Graduate School of Agricultural Science, Tokyo University of Agriculture and Technology, Fuchu, Tokyo 183-8509 Japan; 2 Gene Research Center, Tokyo University of Agriculture and Technology, Fuchu, Tokyo 183-8509 Japan; 3 Department of Entomology and Plant Pathology, Oklahoma State University, Stillwater, OK USA; 4 Faculty of Science and Engineering, Waseda University, Okubo, Shinjuku, Tokyo 169-8555 Japan; 5 Institute of Global Innovation Research (GIR), Tokyo University of Agriculture and Technology (TUAT), Fuchu, Tokyo 183-8509 Japan

**Keywords:** Biotic, Plant molecular biology

## Abstract

The tobacco virus resistance gene *N* contains four introns. Transient expression of transcripts from an *N* transgene containing these introns and driven by the native promoter in the presence of the elicitor of tobacco mosaic virus resulted in its increased expression. The requirement of the native promoter, the elicitor, or the individual introns for enhanced expression of *N* has not been fully studied. Here, we determined that 35S promoter-driven *N* transcript expression could be enhanced in the presence of the four introns regardless of the co-expression of the virus elicitor in tobacco. Function analyses using a series of *N* transgenes with different combination of introns revealed that the presence of intron 1 more so than intron 2 allowed higher accumulation of premature and mature *N* transcripts; however, both introns were important for not only enhanced gene expression but also for induction of cell death in tobacco and induced local resistance to spread of virus in *Nicotiana benthamiana*. Our findings indicate that introns 1 and 2 cooperatively contribute to *N* expression and virus resistance.

## Introduction

Plants defend themselves from pathogen invasions through various defense responses, one of which is the hypersensitive reaction/response (HR) that is accompanied with programed cell death in an infected site. HR is triggered by an interaction of a plant resistance (R) factor with a pathogen avirulence (Avr) factor or an elicitor^1^. *Tobacco mosaic virus* (TMV), genus *Tobamovirus,* is an RNA plant virus that encodes 126 kDa and 183 kDa replicase components, 30 kDa movement protein, and 19 kDa coat protein. TMV rapidly systemically infects tobacco (*Nicotiana tabacum*) but develops only limited local infections on inoculated leaves associated with cell death and tissue necrosis in tobacco cultivars possessing the *N* gene derived from *N. glutinosa*^2^. Tobacco plants with or without the *N* gene are hereafter referred to as NN tobacco and nn tobacco, respectively. The *N* gene encodes a Toll-Interleukin-1 receptor (TIR)-nucleotide binding site (NBS)-leucine rich repeats (LRR) type R factor^3^ that recognizes the helicase domain within the TMV 126/183 kDa proteins as the viral elicitor^4^. HR-like cell death can be induced by transient expression of the viral helicase domain, termed p50, in NN tobacco or by transient co-expression of N and p50 in nn tobacco^5–7^.

Transcription of the endogenous *N* gene is regulated before and after the recognition of the virus elicitor by the N protein; *N* in NN tobacco is expressed at a low level under normal conditions, but up-regulated sharply soon after TMV infection or transient expression of p50^8–10^. This elicitor-responsive gene regulation is considered a strategy for the plant to induce defense responses only when pathogen attacks occur. Recent studies of the upstream regulatory sequence of the *N* gene linked to the green fluorescent protein (GFP) or luciferase (Luc) gene have identified two *cis-*elements important for activation of the *N* promoter in the presence of the N protein and p50^11,12^. However, the activity of the two *cis-*elements does not seem to be sufficient to fully explain elicitor-triggered enhancement of *N* gene expression. Consistent with this idea, transgenic nn tobacco lines that express the *Luc* gene under the control of a 4.1-kb upstream regulatory region of *N* displayed only a limited increase in Luc activity after TMV infection^13^. These results collectively raise the possibility that in addition to the N protein, there is need for sequences further 5′ of the 4.1 kb sequence, or within or 3′ of the *N* gene ORF for maximum elicitor-responsive up-regulation of the *N* gene.

The *N* gene contains four introns (i.e., introns 1, 2, 3, and 4). Limited information is available on roles of these introns in gene expression and virus resistance. Only intron 3 has been shown to have an important role in *N*-mediated resistance. This intron contains an alternative exon that leads to production of an alternatively spliced mRNA encoding Ntr, an N variant with most of the LRR domain truncated^14^. Whereas intron 3 and possibly other introns and the genomic 3′ sequence are necessary for full cell death and resistance to TMV infection^14^, the transient expression of Ntr interferes with induction of cell death and virus resistance^15^. To elucidate roles of the introns of the *N* gene for its function, we developed a transient gene expression system to express the *N* transgene under the control of its upstream regulatory sequences in nn tobacco and demonstrated that the transcript level of the full intron-containing *N* gene, but not an intronless *N* gene, was enhanced as efficiently as the endogenous *N* gene in NN tobacco during co-expression with p50 elicitor^16^. We also determined that expression of a frameshift mutant sequence of the full intron-containing *N* transgene failed to up-regulate its mRNA expression in the presence of the elicitor, but was complemented by a functional N protein supplied *in trans*^16^. In addition, the presence of the introns in the *N* transgene not only elevated the *N* transcript level but also improved the efficiency of cell death induction^16^. These results demonstrate that the introns of the *N* gene and the functional N protein play important roles in elicitor-responsive gene up-regulation and efficient induction of defense responses, but not whether the introns have equal roles in this activity or how they function to increase *N* transcript levels.

Introns of plants often have a positive effect on gene expression, termed intron-mediated enhancement (IME)^17^. In some cases, introns can function as a weak but reproducible promoter containing a transcription initiation site^18,19^ or as a transcriptional enhancer^19–22^. In other cases, gene translation is enhanced by introns without altering the transcript level^23,24^. The mechanisms of IME are still not understood well, but may involve multiple processes during transcription, post-transcription, and translation^25,26^. IME of gene expression influences plant development (within tissues or during transition of growth stages) and the response of plants to environmental abiotic- and biotic-mediated triggers^27–31^. As exemplified by Arabidopsis, introns that are involved in IME are often located in the transcribed sequence or near the promoter, such as the first intron^32^. IME-related introns can function in combination with heterologous promoters such as the CaMV 35S promoter and the nopaline synthase promoter (Nos)^33–35^.

In this study we investigated whether the full intron-containing *N* transgene regulated by the heterologous 35S promoter required expression of the p50 elicitor. In addition, the roles of individual introns in transcript accumulation were examined using agrobacterium to transiently express 35S-driven *N* transgenes containing different combinations of introns. The roles of introns 1 and 2 on *N* gene transcriptional activation, RNA stability, p50-triggered cell death, and virus resistance were studied in detail. Based on findings from these experiments, the roles of introns 1 and 2 for *N* gene expression and virus resistance are discussed.

## Results

### Intron-dependent enhancement of expression of a 35S-driven *N* transgene

**Figure 1:**
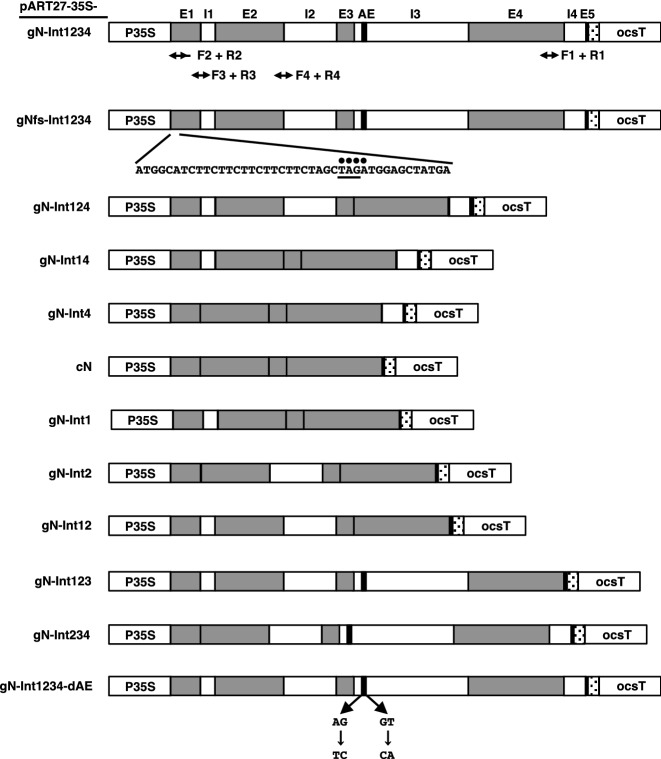
Schematic diagrams of cassettes for transient expression of *N* with a different combination of introns. The pART27-35S-based binary plasmids used in this study carry an expression cassette containing the CaMV 35S promoter (P35S) and the octopine synthase terminator (ocsT), shown in left- and right-most white boxes, respectively. The five exons (E1–E5) and four introns (I1–I4) of the *N* gene are shown in gray and white boxes, respectively. E1 includes a coding sequence following a 5′-UTR sequence derived from the vector plasmid. E5 contains a coding sequence linked to a double hemagglutinin tag-encoding sequence shown by a dotted box, followed by a 3′-UTR sequence derived from the vector plasmid. A black bar within I3 indicates the alternative exon (AE) of the *N* gene. Four dots and a single underline in pART27-35S-gNfs-Int1234 indicate a four-nucleotide insertion at an XbaI recognition site and a stop codon caused by the insertion, respectively. Nucleotide substitutions of the splicing motif for alternative splicing (AG–TC and GT–CA) are indicated in pART27-35S-gN-Int1234-dAE. Bi-directional arrows below pART27-35S-gN-Int1234 indicate the regions amplified by the indicated primer sets (F1 + R1, F2 + R2, F3 + R3, and F4 + R4).

**Figure 2:**
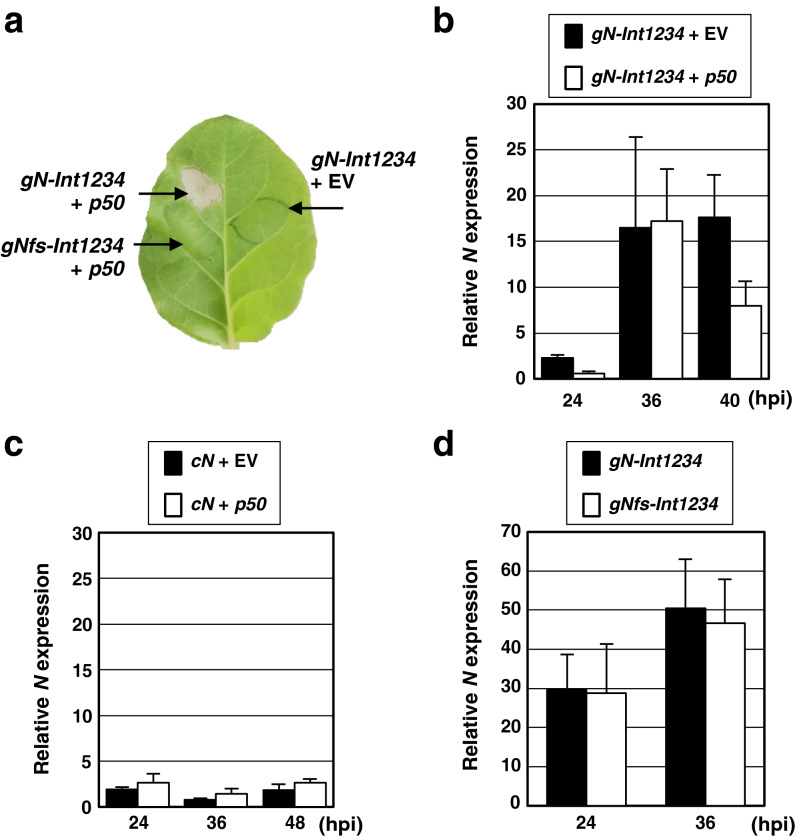
Intron-dependent enhanced expression of 35S-driven *N* in the presence or absence of elicitor, p50. (**a**) Agrobacterium transformants carrying a plasmid containing *gN-Int1234* or *gNfs-Int1234* were infiltrated with those carrying *p50* to nn tobacco. In a control experiment for *gN-Int1234*, agrobacteria carrying the empty vector (EV) were used. A photo was taken at 7 days post-infiltration. (**b**, **c**) Agrobacterium transformants carrying a plasmid of *gN-Int1234* (**b**) or *cN* (**c**) were infiltrated with those carrying *p50* or EV into nn tobacco. Transcript levels for *gN-Int1234* and *cN* were analyzed by RT-qPCR using the F1 + R1 primer set at 24, 36, and 40 (for *gN-Int1234*) or 48 (for *cN*) h post-infiltration (hpi). The transcript levels of *gN-Int1234* + EV and *cN* + EV were adjusted to 1 with levels for other treatments relative to these values. (**d**) The transcript levels of *gN-Int1234* and *gNfs-Int1234* in the absence of elicitor expression, determined by RT-qPCR with the F1 + R1 primer set at 24 and 36 hpi, are shown. Transcript levels of *actin* were used for normalization of all values in (**b**–**d**). Values are the means ± SE of three independent biological replicates.

To investigate the influence of the four introns of the *N* gene on its expression under the control of the CaMV 35S promoter, we used two expression constructs that carried the genomic sequence with the four introns (*gN-Int1234*) or the intron-less cDNA sequence (*cN*) of the *N* gene (Fig. 1). Note that the sequences of exon 1 and exon 5 in *gN-Int1234* lack the 5′- and 3′-UTR sequence of the *N* gene, respectively (Fig. 1). In this study, agrobacterium transformants with pART27 empty vector (EV) or each of its derivatives were used for transient expression experiments by an agroinfiltration method. Tobacco leaves expressing *gN-Int1234* or *cN* transiently, with or without co-expression of the *p50* cDNA, were subjected to reverse transcription-quantitative real time PCR (RT-qPCR) analysis at 24, 36, and 40 h post-infiltration (hpi; for *gN-Int1234*) or 48 hpi (for *cN*). A primer set of F1 and R1 was used to amplify part of exon 4 of transcripts from an *N* transgene (Fig. 1). Severe cell death associated with necrosis was observed in tissues co-expressing *gN-Int1234* with *p50* but not those expressing *gN-Int1234* alone (Fig. 2a). The necrosis progressed so quickly that tissue was harvested at 40 hpi instead of 48 hpi to avoid confounding necrosis effects on transcription. Interestingly, in the presence or absence of *p50* co-expression the level of *gN-Int1234* transcript was increased equally from 12 to 36 hpi and maintained high levels for both treatments through 40 hpi (Fig. 2b). In contrast, the *cN* transcripts with and without the *p50* co-expression constantly accumulated at low levels (Fig. 2c).

To examine whether the N protein was involved in the observed enhancement of the *gN-Int1234* expression, a *gN-Int1234* variant carrying a frameshift mutation in the coding sequence of exon 1 (*gNfs-Int1234*) was made. Co-expression of *gNfs-Int1234* and *p50* induced no cell death, suggesting that no functional N protein was synthesized (Fig. 2a). Then, RT-qPCR was conducted using tobacco leaves expressing *gN-Int1234* or *gNfs-Int1234* without the *p50* co-expression and it was determined that *gN-Int1234* and *gNfs-Int1234* produced similar levels of transcript at both 24 and 36 hpi (Fig. 2d), indicating that N protein production is dispensable for high accumulation of these intron-containing transcripts. These collective results demonstrated that the expression of the 35S-driven *gN-Int1234* transgene could be enhanced in the presence of the four introns regardless of the co-expression of the viral elicitor and the N protein.

### Both of introns 1 and 2 are required and sufficient for enhanced expression of *N* transgene

**Figure 3:**
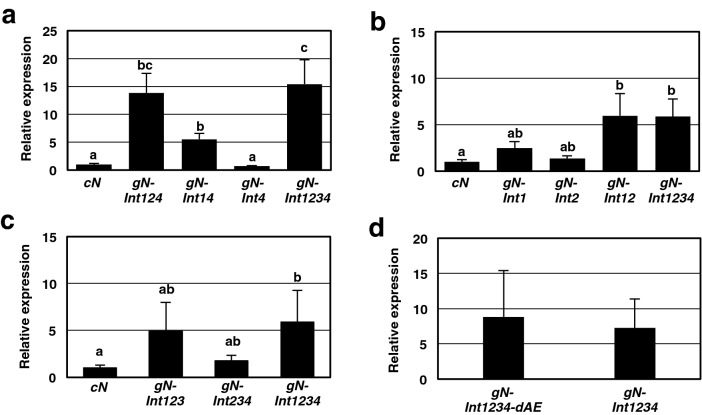
Involvement of introns 1 and 2 in the enhanced expression of the 35S-driven *N* transgene. (**a**–**d**) Agrobacterium transformants carrying a plasmid for the indicated *N* transgene were infiltrated to nn tobacco. The levels of *N* transcript were analyzed by RT-qPCR with F1 + R1 primer set at 36 h post-infiltration (hpi). The transcript levels of *cN* were adjusted to 1 with levels for other treatments relative to these values. The transcript levels of *actin* were used for normalization of all values. Values are the means ± SE of three independent biological replicates. Data in (**a**–**c**) were analyzed by the two-way ANOVA test followed by the Tukey–Kramer test (p < 0.05). Different letters above the bars indicate the means are significantly different from each other at the 0.05 level. Absence of letters above bars in (**d**) indicates no significant difference between values.

To determine which introns of the four contribute to the enhanced expression of *gN-Int1234*, a deletion series of *N* transgenes that lacked intron 3 (*gN-Int124*), introns 2 and 3 (*gN-Int14*), or introns 1, 2, and 3 (*gN-Int4*) were constructed (Fig. 1) and expressed transiently in nn tobacco. RT-qPCR analysis using the primer set F1 + R1 to quantify transcripts at 36 hpi revealed that *gN-Int124* and *gN-Int4* were expressed at levels similar to those of *gN-Int1234* and *cN*, respectively, while the transcript level of *gN-Int14* was approximately one-third that of *gN-Int1234* (Fig. 3a). Thus, it was possible that introns 1 and 2 both were important for enhanced expression of *gN-Int1234*.

To test the importance of introns 1 and 2 for *N* gene expression, another series of modified *N* genes carrying intron 1 (*gN-Int1*), intron 2 (*gN-Int2*), or both introns 1 and 2 (*gN-Int12*) were constructed (Fig. 1) and examined for their transcript levels at 36 hpi. As shown in Fig. 3b, the transcript level of *gN-Int1* was nearly half that of *gN-Int1234* while the transcript level of *gN-Int2* was only slightly higher than that of *cN*. This result suggested that the presence of intron 1 alone had the ability to increase *N* transcript levels. However, the transcript level of *gN-Int12* was greater than *gN-Int1* alone and similar to *gN-Int1234* transcript levels (Fig. 3b), showing that the concomitant presence of just the two introns was sufficient to express *N* at the level observed for *gN-Int1234*. Further support for the requirement of introns 1 and 2 together for maximum *N* expression came from analysis of another *N* gene lacking intron 1 only (*gN-Int234*). *gN-Int234* had less than one-third the transcript level of *gN-Int1234* (Fig. 3c).

Lastly, we examined the transcript levels of *gN-Int123* and *gN-Int1234-dAE*. *gN-Int123* carries introns 1, 2, and 3 but not intron 4 while *gN-Int1234-dAE* contains intron 3 with nucleotide substitutions in the GT-AG motif of the alternative exon (Fig. 1). Transcript levels of both of *gN-Int123* and *gN-Int1234-dAE* were similar to those of *gN-Int1234* at 36 hpi (Fig. 3c,d). These were consistent with findings that *gN-Int4* did not increase transcript levels above those observed for *cN* (Fig. 3a) and that *gN-Int12* and *gN-Int123* accumulated their transcripts to levels similar to *gN-Int1234* (Fig. 3b,c). These results indicate that intron 4 and intron 3-mediated alternative splicing have no influence on enhancing expression of *gN-Int1234*.

### Greater ***N*** transcript in the presence of introns 1 and 2 is positively correlated with the presence of more ***N*** transcript containing exon 4 toward the 3′ end of the open reading frame

**Figure 4:**
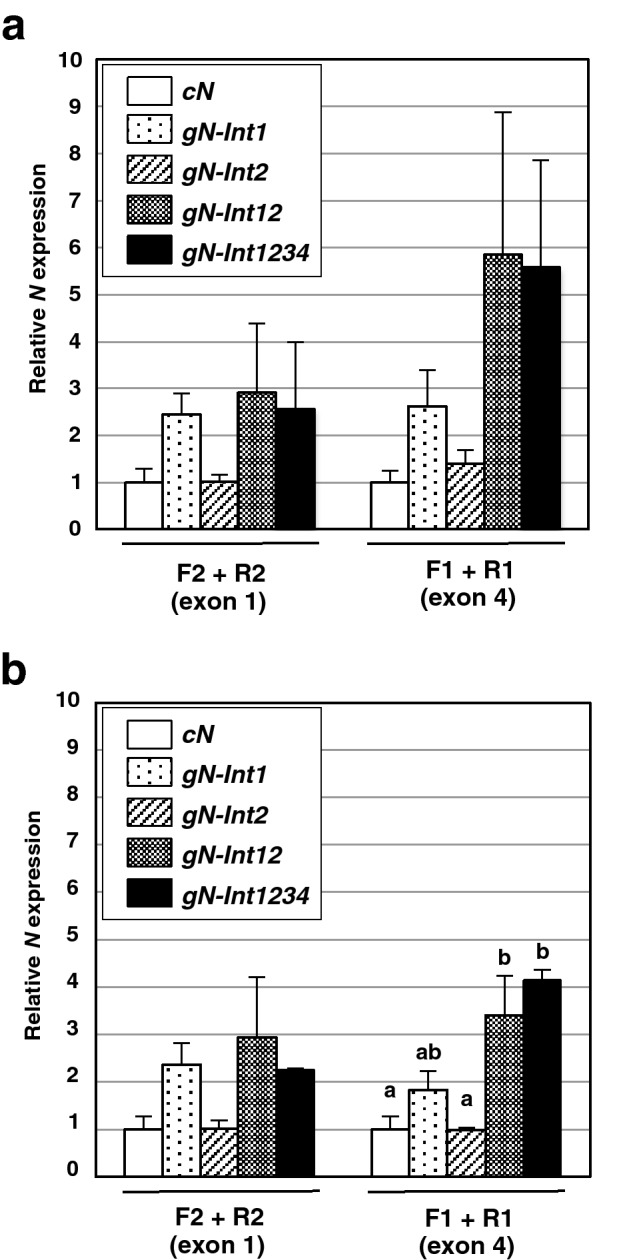
Introns 1 and 2 cooperatively contribute to produce more *N* transcripts containing the 3-proximal region within the open reading frame. Agrobacterium transformants carrying a plasmid for *cN*, *gN-Int1*, *gN-Int2*, *gN-Int12*, or *gN-Int1234* were infiltrated to nn tobacco. The 5′- and 3′-proximal regions of the *N* transcript open reading frame at 36 (**a**) and 48 (**b**) h post-infiltration were quantified by RT-qPCR with the F2 + R2 primer set amplifying a portion of exon1 and the F1 + R1 primer set amplifying a portion of exon 4. The transcript levels of *cN* were adjusted to 1 with levels for other treatments relative to these values. Transcript levels of *actin* were used for normalization of all values in (**a**, **b**). Values are the means ± SE of three independent biological replicates. Data in (**a**) and those of F1 + R1 in (**b**) were analyzed by the two-way ANOVA test followed by the Tukey–Kramer test (p < 0.05). Different letters above the bars indicate the means are significantly different from each other at the 0.05 level. Absence of letters in (**a**) indicates no significant difference between treatment means obtained through amplification with either F1 + R1 or F2 + R2. Note that variances around the means for data of F2 + R2 in (**b**) were not homogeneous. For the data set, the non-parametric Steel–Dwass one-way analysis did not show significant differences in the means.

Given that the presence and absence of intron(s) can influence stability of transcripts^36^, we hypothesized that introns 1 and 2 might influence the integrity of the *N* gene transcript. To address this hypothesis, we designed an additional primer set (F2 and R2) to amplify a portion of exon 1 in the *N* transcript and carried out RT-qPCR to compare levels of transcripts amplified by F2 + R2 (representing a portion of exon 1) with those of transcripts amplified by F1 + R1 (representing a portion of exon 4). Transcript levels of *cN*, *gN-Int1*, *gN-Int2*, *gN-Int12*, and *gN-Int1234* were examined at 36 and 48 hpi. Consistent with the result shown in Fig. 3b, RT-qPCR using the F1 + R1 primer set demonstrated that the transcript levels of *gN-Int1* or *gN-Int12* were, respectively, nearly half of or comparable to that of *gN-Int1234* and that *gN-Int2* accumulated its transcripts at levels similar to those of *cN* (Fig. 4a,b). For primer set F2 + R2, the transcript levels of *gN-Int1* and *gN-Int12* were nearly comparable to *gN-Int1234* transcript levels (Fig. 4a,b). These results suggested that the 3′-proximal region of transcripts of *gN-Int1* and *gN-Int2* as well as *cN* might be less stable than those of *gN-Int12* and *gN-Int1234*. Thus, both introns 1 and 2 might contribute cooperatively to the production of more *N* transcripts with the 3-proximal region.

### The presence of intron 1 but not intron 2 in an *N* transgene led to enhanced accumulation of premature transcripts

**Figure 5:**
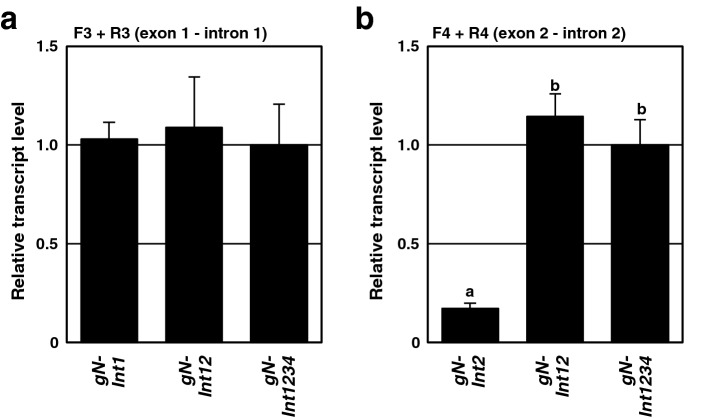
Involvement of intron 1, but not intron 2, in the enhanced accumulation of premature transcripts. Agrobacterium transformants carrying a plasmid for *gN-Int1*, *gN-Int2*, *gN-Int12*, or *gN-Int1234* were infiltrated to nn tobacco. Intron-containing premature transcripts from these *N* transgenes at 48 h post-infiltration were quantified by RT-qPCR with the F3 + R3 primer set amplifying a portion of the exon1—intron 1 junction (**a**) or the F4 + R4 primer set amplifying a portion of the exon 2 – intron 2 junction (**b**). The transcript levels of *gN-Int1234* were adjusted to 1 with levels for other treatments relative to these values. Transcript levels of *actin* were used for normalization of values in (**a**, **b**). Values are the means ± SE of three biological replicates. Data in (**a**, **b**) were analyzed by the two-way ANOVA test followed by the Tukey–Kramer test (p < 0.05). Different letters above the bars in (**b**) indicate that the means are significantly different at the 0.05 level. Absence of letters in (**a**) indicates no significant difference between values.

Transcript levels for *gN-Int1* were always greater than those for *gN-Int2* regardless of when after infiltration the analysis was done or where on the open reading frame RT-qPCR amplifications were conducted, even though not statistically significant (Figs. 3 and 4). The above results raised a possibility that introns 1 and 2 may have different effects on accumulation of premature transcripts of the *N* transgenes. To test the possibility, we determined levels of premature transcripts of *gN-Int1*, *gN-Int2*, *gN-Int12*, and *gN-Int1234* by using primer sets designed to amplify the sequence linking exon 1 with intron 1 (F3 and R3), or exon 2 with intron 2 (F4 and R4) (Fig. 1). As shown in Fig. 5a, the levels of transcripts that were amplified by F3 + R3 were comparable between *gN-Int1*, *gN-Int12*, and *gN-Int1234* at 48 hpi, implying that their premature transcripts containing intron 1 were produced at the same efficiencies. In contrast, *gN-Int2* transcripts amplified by F4 + R4 were detected at a significantly lower level compared with those of *gN-Int12* and *gN-Int1234*, both of which showed similar transcript levels (Fig. 5a,b), indicating that intron 2 is not enough to enhance the accumulation of intron 2-containing premature transcripts. These results suggested that the presence of intron 1 but not intron 2 of the *N* gene is involved in activation of transcription from an *N* transgene and/or protection of premature transcripts from degradation.

### Cooperative roles of introns 1 and 2 of the *N* gene in p50-triggered cell death in nn tobacco

**Figure 6:**
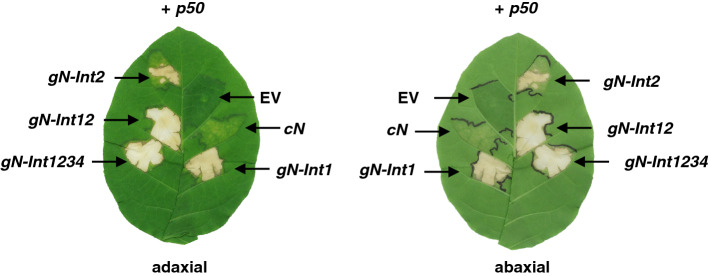
Effects of introns 1 and 2 on p50-triggered, N-mediated cell death in nn tobacco. Agrobacterium transformants carrying the indicated *N* transgene were infiltrated with those carrying *p50* into nn tobacco. As a control, agrobacteria carrying the empty vector (EV) were used. Representative photos of an adaxial (left) and abaxial (right) side from an infiltrated leaf at 11 dpi are shown.

To investigate relationships between the presence of specific introns and elicitor-triggered cell death, we selected some modified *N* genes (i.e., *cN*, *gN-Int1234*, *gN-Int1*, *gN-Int2*, and *gN-Int12*) and co-expressed them transiently with the *p50* cDNA in nn tobacco. Agrobacterium containing no insert in the plasmid vector (EV) was used as a negative control. Agrobacterium-infiltrated plants were kept at 25 °C, the same temperature used for RT-qPCR analyses. Cell death progress was evaluated based on visual observation and the following scoring criteria: 0, no visible signs of cell death; 1, change from a smooth to irregular surface of the abaxial epidermis; 2, signs of withering and clearing of leaf tissue; 3, formation of gray-to-brownish necrotic lesion; 4, formation of entirely chalky white necrosis. Under this moderate temperature condition, the expression of *cN* rarely induced visible necrosis (Fig. 6 and Table 1). The expression of the other four *N* genes induced visible necrotic lesions, but the progression toward and degree of cell death for each were sometimes unique. The tissues expressing *gN-Int12* and *gN-Int1234* quickly and similarly developed chalky white necrotic lesions by 5 dpi, while those expressing *gN-Int1* and *gN-Int2* predominately formed gray-to-brownish lesions through 7 dpi (Fig. 6a,b). These results showed that full necrosis phenotype required both introns 1 and 2 to be present in the *N* transgene. However, the presence of either intron was individually enough to initiate cell death after interaction with the virus elicitor. The difference in transcript levels between *gN-Int1* and *gN-Int2* (Figs. 3 and 4) did not affect the speed of appearance and phenotype of cell death.

**Table 1:** Effects of introns 1 and 2 during *N*-mediated progression to cell death within tobacco leaves expressing p50 elicitor.

Expressed transgene^a^	Leaf number	Days after infiltration^b^						
		1	2	3	4	5	6	7
EV	#1	0	0	0	0	0	0	0
	#2	0	0	0	0	0	0	0
	#3	0	0	0	0	0	0	0
	#4	0	0	0	0	0	0	0
	#5	0	0	0	0	0	0	0
	#6	0	0	0	0	0	0	0
*cN*	#1	0	0	1	1	1	1	1
	#2	0	0	0	1	1	1	1
	#3	0	0	0	0	1	1	1
	#4	0	0	0	0	1	1	1
	#5	0	0	0	0	1	1	1
	#6	0	2	3	3	3	3	3
*gN-Int1*	#1	0	1	2	3	3	3	3
	#2	0	2	2	3	3	3	3
	#3	0	2	2	3	3	3	3
	#4	0	2	2	3	3	3	3
	#5	0	2	2	3	3	3	3
	#6	1	2	3	3	4	4	4
*gN-Int2*	#1	0	1	2	3	3	3	3
	#2	0	1	2	3	3	3	3
	#3	0	2	2	3	3	3	3
	#4	0	2	2	3	3	3	3
	#5	0	2	2	3	3	3	3
	#6	0	2	3	3	4	4	4
*gN-Int12*	#1	0	2	2	3	3	3	3
	#2	0	2	3	3	4	4	4
	#3	0	2	3	3	4	4	4
	#4	0	2	3	3	4	4	4
	#5	0	2	3	3	4	4	4
	#6	1	2	3	3	4	4	4
*gN-Int1234*	#1	0	2	2	3	3	3	3
	#2	1	2	3	3	4	4	4
	#3	0	2	3	3	4	4	4
	#4	1	2	3	3	4	4	4
	#5	1	2	3	3	4	4	4
	#6	2	2	3	3	4	4	4

^a^Each of five *N* transgenes (*cN*, *gN*-*Int1*, *gN*-*Int2*, *gN*-*Int12*, and *gN*-*Int1234*) was expressed together with *p50* in the same tobacco leaf (#1 to #6). Agrobacterium with empty vector (EV) was used as a negative control.

^b^Scoring criteria for cell death progress: 0, no visible signs of cell death; 1, change from a smooth to irregular surface of the abaxial epidermis; 2, signs of withering and clearing of leaf tissue; 3, formation of gray-to-brownish necrotic lesion; 4, formation of entirely chalky white necrosis.

### Cooperative roles of introns 1 and 2 in *N*-mediated virus resistance in *Nicotiana benthamiana*

To further investigate roles of introns 1 and 2 in virus resistance, we carried out an agrobacterium-mediated inoculation experiment using *cN*, *gN-Int1234*, *gN-Int1*, *gN-Int2*, and *gN-Int12*, and an infectious ToMV mutant clone encoding ER-targeting GFP instead of coat protein (ToMV-erGFP)^15^. *Nicotiana benthamiana* was used because it has been shown to induce *N*-mediated virus resistance without visible cell death^15,37^. Changes in the size of infection foci indicated by GFP fluorescence were monitored under a fluorescent microscope.

We firstly determined percentages of a total fluorescent area, representing accumulation of ToMV-erGFP, over a co-infiltrated area with *cN* or *gN-Int1234* from 4 to 6 dpi. Fluorescent areas for leaf tissue infiltrated with *gN-Int1234* were significantly smaller than those infiltrated with *cN* during the observation period, indicating that virus resistance mediated by *gN-Int1234* was stronger than that mediated by *cN*. On the other hand, the fluorescent areas for *cN* were comparable in size to those present after infiltration with agrobacterium transformants with no insert (EV) and the virus genome throughout the experimental time frame (Supplemental Fig. 1).

**Figure 7﻿:**
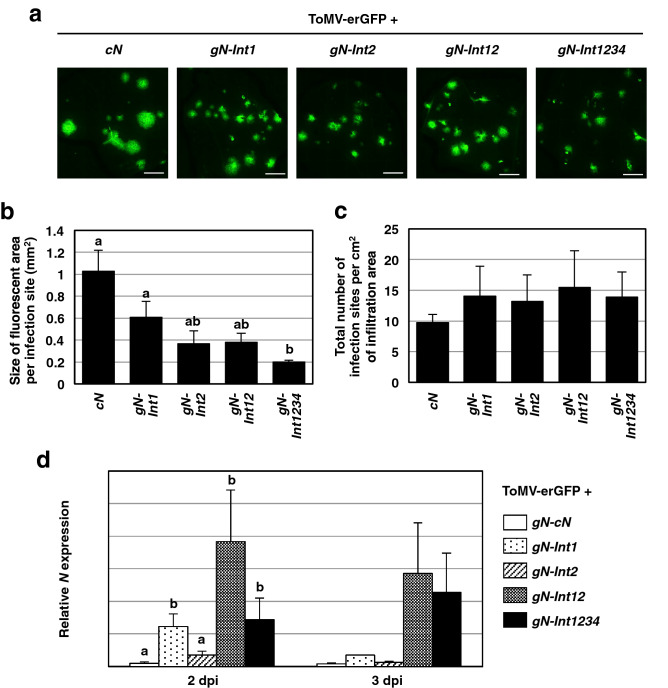
Effects of introns 1 and 2 on N-mediated virus resistance in *Nicotiana benthamiana*. (**a**) Agrobacterium transformants carrying the indicated *N* transgene were infiltrated with those carrying the infectious ToMV-erGFP clone into *N. benthamiana*. Representative fluorescent images taken at 5 days post-infiltration are shown. Bar = 2.5 mm. (**b**, **c**) The mean size of fluorescent areas per infiltration site and the total number of infection loci per cm^2^ were determined for each *N* transgene. (**d**) *N* transcript levels were analyzed by qRT-PCR with the F1 + R1 primer set at 2 and 3 days post-infiltration (dpi). Transcript levels of *actin* were used for normalization of values. Values of (**b**), (**c**) or (**d**) are the means ± SE of, respectively, six or three biological replicates. Data in (**b**) or (**c**, **d**) (for 2 dpi) were analyzed, respectively, by the Steel–Dwass test (p < 0.05) or a two-way ANOVA test followed by the Tukey–Kramer test (p < 0.05). Different letters above the bars in (**b**) and (**d**) (2 dpi) indicate that the means are significantly different from each other at the 0.05 level. Absence of letters above bars in (**c**) and in (**d**) (3 dpi) indicates no significant difference between values.

ToMV-erGFP infected leaf areas that expressed each of the five *N* transgenes were further examined at 5 dpi to determine the size and number of individual spots (spreading local infection loci) (Fig. 7). The mean sizes of individual virus-mediated fluorescent spots for *cN*, *gN-Int1*, *gN-Int2*, *gN-Int12,* and *gN-Int1234* were, respectively, approximately 1.0, 0.6, 0.4, 0.4, and 0.2 mm^2^ (Fig. 7b). These results suggested that the expression of *gN-Int1*, *gN-Int2*, *gN-Int12* and *gN-Int1234* led to progressively stronger virus resistance compared with *cN*, although statistical significance was obtained only between *gN-Int1234* and *cN* or *gN-Int1* (Fig. 7b). On the other hand, the total numbers of infection sites per area were similar among the five different *N* transgenes tested (Fig. 7c), suggesting that the effect of their transient expression is primarily on virus intercellular movement rather than preventing initial infection.

Similar to the results from the transient expression experiments in tobacco (Figs. 3 and 4), RT-qPCR analysis using the F1 + R1 primer set showed that *gN-Int1* and *gN-Int12* as well as *gN-Int1234* infiltrated tissue displayed high levels of transcripts in comparison with tissue infiltrated with *cN* at both 2 and 3 dpi (Fig. 7d). Tissue infiltrated with g*N-Int2,* however, accumulated low levels of its transcript at 2 and 3 dpi, similar to *cN* (Fig. 7d). Collectively, these results indicated that introns 1 and 2 of the *N* gene could function cooperatively in enhanced gene expression as well as virus resistance in *N. benthamiana*, and also showed that the transcript levels of the modified *N* genes tested, such as *gN-Int1* and *gN-Int2*, did not perfectly correlate with the degree of virus resistance observed for each modified *N* (i.e. low expression of *gN-Int2* provided as good protection as higher expression of *gN-Int1*).

### Compilation analysis demonstrated statistically significant increases in transcript levels of *gN-Int1*, *gN-Int12*, and *gN-Int1234* compared with those of *cN* and *gN-Int2*

Considering that five independent experiments using the same F1 + R1 primer set (i.e., Figs. 3b, 4a,b, and 7d) were conducted at times from 36 to 72 hpi to compare transcript levels between *cN*, *gN-Int1*, *gN-Int2*, *gN-Int12*, or *gN-Int1234*, it was important to utilize the power of these repetitive experiments to support the observation that transcript levels for *gN-Int1*, *gN-Int12*, and *gN-Int1234* were always greater than those for *cN* and *gN-Int2*. Utilizing the mean value of *cN* for normalization of other transcription levels in each experiment and analyzing all normalized data by the Steel–Dwass non-parametric test, it was determined that transcript levels of *gN-Int1*, *gN-Int12*, and *gN-Int1234* were significantly greater than those of *cN* and *gN-Int2* (Supplemental Fig. 2).

## Discussion

We have recently demonstrated that the elicitor-responsive up-regulation of the *N* gene mRNA depends on both the presence of the four introns and the expression of the functional N protein^16^. Here, using the full intron-containing *gN-Int1234* and intron-less *cN* genes with the 35S promoter instead of the native *N* gene promoter (NP2.3), we show that the presence of these introns can enhance the transcript level either with or without elicitor expression. In addition, our finding that the frameshift mutant *N* gene, *gNfs-Int1234,* exhibited as high transcript level as *gN-Int1234* further supports the conclusion that the interaction between N and p50 is unnecessary for the intron-mediated up-regulation of the *N* transgene expression under the 35S promoter. The difference in the requirement of the N-p50 interaction between the 35S promoter and NP2.3 may be due to the difference in their basal promoter activities. Unlike the 35S promoter that is known to be constitutively active, it is possible that NP2.3 is repressed under normal conditions based on findings showing that *N* and *gN-int1234* are expressed at low levels prior to TMV infection or elicitor expression^8–10,16^. Our findings indicate that the effects of the virus elicitor and the introns on *N* expression can be unlinked from each other, independently increasing *N* mRNA levels.

Results from experiments using g*N-Int1234* and its derivatives with different combinations of introns 1 to 4 demonstrated that the co-presence of introns 1 and 2 alone was necessary and sufficient to increase transcript levels to those observed for *gN-Int1234* (Figs. 3, 4 and 7). However, our results indicate that *N* with intron 1 alone (*gN-Int1*), but not intron 2 alone (*gN-Int2*), has the ability to increase transcript levels, though less efficiently than *gN-Int1234,* suggesting that introns 1 and 2 have some different functions in regulating *N* gene expression (discussed below). In addition, considering that the *N* transgenes lacking intron 3 (*gN-Int124*), intron 4 (*gN-Int123*), or the alternative splicing motif sequences (*gN-Int1234-dAE*) accumulated their transcripts to the levels comparable to that of *gN-Int1234*, we assume that introns 3 and 4 and also alternative splicing are not essential for induction of high transcript levels. Thus, among the four introns of the *N* gene, the combination of introns 1 and 2 may play a major and cooperative role in enhanced gene expression under the 35S promoter.

In an effort to understand what species of mRNA was being induced and accumulating during *N* expression, it was interesting to find that transcripts for *gN-Int1* were similar in level to those of *gN-Int12* and *gN-Int1234* when primers amplifying the 5′-proximal region of the *N* mRNA were used in RT-qPCR assays but less when primers amplifying the 3′-proximal region were used (Fig. 4). Thus, the 3′-proximal region of the transcripts of *gN-Int1* may be less stable than those of *gN-Int12* and *gN-Int1234* while their 5′-proximal regions are likely to be equally stable. In addition, similar levels of both proximal regions between *cN* and *gN-Int2* at both 36 and 48 hpi support our hypothesis that intron 2 alone does not enhance transcript level. Furthermore, our comparison of the levels of intron-containing transcripts has shown that *gN-Int1* but not *gN-Int2* can produce its premature transcripts at a level comparable to those of *gN-Int12*, and *gN-Int1234* (Fig. 5). These results indicate that that the presence of intron 2 alone is insufficient to enhance accumulation of premature transcripts. Intron 1-mediated enhancement of premature transcript accumulation may involve the activation of transcription from the 35S promoter and/or protection of the transcripts from degradation. Also, although intron 2 does not increase premature transcripts or total transcript, it appears necessary, with intron 1, to enhance accumulation of transcript containing exon 4 (i.e. transcript containing 3′ exons). Collectively, it is possible that the high transcript levels of the 35S-driven *N* transgenes containing both introns 1 and 2, such as *N-Int12* and *N-Int1234*, may result from multiple effects involving transcriptional activation and/or transcript stabilization by the two introns.

The co-expression of *N-Int12* or *N-Int1234* with *p50* caused complete cell death (chalky white necrosis) while the tissues co-expressing *gN-Int1* or *gN-Int2* with *p50* developed slightly delayed and attenuated cell death (gray-to-brownish necrosis). In contrast, as shown in the case of *cN*, the expression of the intron-less transgene resulted in a very limited cell death. Thus for necrosis induction, as for transcript induction, introns 1 and 2 together are required for maximum effect while either alone induces only partial effect. Also, introns 3 and 4 are dispensable for inducing the necrosis response. It is important to note, however, that the ability of introns 1 and 2 to give similar necrosis response patterns without identical transcript levels (compare Figs. 3 and 4 with Fig. 6 and Table 1) indicates that the two introns function differently to induce cell death or that *N* gene transcript levels only need to reach a certain threshold to induce the slower cell death phenotype. Further research is necessary to understand this loss of correlation between transcript level and cell death induction.

In *N*. *benthamiana*, a positive correlation between increased transcript level and, in this case, heightened resistance to virus spread (but not virus infectivity) was observed (Fig. 7). This result again shows the requirement of introns 1 and 2 for an *N* transgene to mimic effects observed for *gN-Int1234* containing all four introns. However, we speculate that intron 2 of *N* may have a more crucial role at the translation level than intron 1 in exhibiting the inhibitory effect on virus movement because transcript levels of *gN-Int2* were lower than those of *gN-Int1* and rather close to those of *cN*. It is possible that intron 2 may contribute to increasing translation efficiency, leading to a higher production of the R factor. It is known in another system that greater R protein accumulation is associated with a greater resistance phenotype^38^. Utilizing a C-terminally tagged HA epitope would allow us to compare the levels of the N protein among the *N* transgenes. However, we have not yet succeeded to establish the immunological detection system for the HA-tagged N protein reproducibly and stably due to its high molecular weight. Future analysis at the protein level will elucidate how introns 1 and 2 are individually or cooperatively involved in regulating translation efficiency.

We determined that *gN-Int4* did not induce p50-triggered cell death in nn tobacco despite having a transcript level similar to that of *cN*. It is possible that *gN-Int4* mRNA does not translate well or is unstable, either outcome leading to less N protein that can interact with the elicitor. Consistent with this idea, our additional experiments have revealed that the transcripts from g*N-Int4* are prematurely terminated within an exon 4 sequence via alternative polyadenylation (in preparation). A possible role of introns 3 and 4 in RNA processing and elicitor-triggered virus resistance will be discussed elsewhere.

Our findings in this study provide a starting point to further investigate how introns 1 and 2 function as IME elements in the elicitor-responsive up-regulation of the *N* gene in NN tobacco. Transient expression analysis using NP2.3-driven *N* transgenes with different combinations of introns 1 and 2 is ongoing. These and other analyses will further unveil IME function of the introns of the *N* gene to regulate its gene expression for effective induction of virus resistance. As for the *N* gene, introns of many genes have been reported to be involved in gene expression regulation affecting transcription, post-transcription, and/or translation^26^. However, the mechanism and regulation of IME in plants is not fully resolved yet, making it difficult to properly utilize introns to usefully alter gene expression in plants. Further mechanistic study of IME of *N* gene expression not only may allow proper use of the *N* gene introns for enhanced virus resistance in modified plants, but lead to greater understanding of methods to utilize introns generally for agronomic benefit.

## Methods

### Plant material and growth

Experiments were carried out with tobacco (*Nicotiana tabacum* cv Samsun NN and nn) and *N. benthamiana*. Seeds of tobacco and *N. benthamiana* were kindly gifted from Dr. Hideki Takahashi at Tohoku University and Dr. Tetsuo Meshi at the National Agriculture and Food Research Organisation, respectively. Plants were grown as described previously^39^. 7- to 9-week-old plants were selected for all experiments.

### Construction of binary plasmids

pART27-35S-gN-Int1234, pART27-35S-gN-Int124, pART27-35S-gN-Int14, and pART27-35S-gN-Int4:pDONR/Zeo-N is an entry plasmid containing the *N* gene open reading frame^16^. pDONR/Zeo-gN-Int4, pDONR/Zeo-gN-Int14, pDONR/Zeo-gN-Int124, and pDONR/Zeo-gN-Int1234 were constructed in order by inserting the sequence of intron 4, intron 1, intron 2, and intron 3 into pDONR/Zeo-N, pDONR/Zeo-gN-Int4, pDONR/Zeo-gN-Int14, and pDONR/Zeo-gN-Int124, respectively, through standard restriction digestion and ligation techniques^16^. A Gateway cloning technology-based recombination through LR reaction (Invitrogen, Carlsbad, CA, USA) was done between the pART27-35S-GWB-DHA destination vector containing the CaMV 35S promoter expression cassette^7^ and pDONR/Zeo-gN-Int4, pDONR/Zeo-gN-Int14, pDONR/Zeo-gN-Int124, or pDONR/Zeo-gN-Int1234, generating pART27-35S-gN-Int4, pART27-35S-gN-Int14, pART27-35S-gN-Int124, pART27-35S-gN-Int1234.

pART27-35S-gN-Int123 and pART27-35S-gN-Int234:pDONR/Zeo-gN-Int1234 was digested with BglII and EcoRV or StuI and XbaI to remove the DNA sequence containing intron 4 or intron 1, respectively. The respective digested plasmids were ligated with the corresponding small fragment from pDONR/Zeo-N that was digested with BglII and EcoRV or StuI and XbaI. The resultant plasmids named pDONR/Zeo-gN-Int123 and pDONR/Zeo-gN-Int234 were recombined with pART27-35S-GWB-DHA through LR reaction to obtain pART27-35S-gN-Int123 and pART27-35S-gN-Int234, respectively.

pART27-35S-gN-Int1:pDONR/Zeo-gN-Int14 was digested with BglII and EcoRV to remove a DNA sequence containing intron 4 and ligated with a corresponding small fragment from pDONR/Zeo-N that was digested with BglII and EcoRV. The resultant plasmid named pDONR/Zeo-gN-Int1 was recombined with pART27-35S-GWB-DHA through LR reaction to obtain pART27-35S-gN-Int1.

pART27-35S-gN-Int2:pDONR/Zeo-gN-Int234 was digested with EcoRV and SalI to remove the DNA sequence containing introns 3 and 4 and ligated with the corresponding small fragment from pDONR/Zeo-N that was digested with EcoRV and SalI. The resultant plasmid named pDONR/Zeo-gN-Int2 was recombined with pART27-35S-GWB-DHA through LR reaction to obtain pART27-35S-gN-Int2.

pART27-35S-gN-Int12:pDONR/Zeo-gN-Int123 was digested with EcoRV and SalI to remove the DNA sequence containing intron 3 and ligated with the corresponding small fragment from pDONR/Zeo-N that was digested with EcoRV and SalI. The resultant plasmid named pDONR/Zeo-gN-Int12 was recombined with pART27-35S-GWB-DHA through LR reaction to obtain pART27-35S-gN-Int12.

pART27-35S-gNfs-Int1234:pART27-35S-gN-Int1234 was digested with XbaI, filled-in with the Klenow fragment of DNA polymerase I (Takara Bio, Shiga, Japan), and self-ligated to obtain pART27-35S-gNfs-Int1234 that has four extra nucleotides causing a frameshift mutation and a premature termination codon (Fig. 1).

pART27-35S-gN-Int1234-dAE: PCR was first carried out using pDONR/Zeo-gN-Int1234 as a template DNA and a primer set, gN/F2 (5′-CCAACATGGAAAACTTATAAACTGG-3′) and N/dAE/R1 (5′-GAAAGAGGGAGATGGAATTCTTATTGAATTTTGGGGCGATTTACAATGGGCAAAGACCCCTC-3′) or N/dAE/F1 (5′-AATTCAATAAGAATTCCATCTCCCTCTTTCTCTGCAATATTGTTCTTCTTGATTTCTTGTTT-3′) and Nlrr/R03 (5′-AGTTCTGGTAGCTGTGTAAG-3′) to obtain an 883 or 2565 bp fragment, respectively. The former and latter contain nucleotide substitutions from AG to TG and GT to CA, respectively, which resulted in disruption of the splicing motif for alternatively spliced exon within intron 3 (Fig. 1). Heteroduplex-mediated DNA amplification using the two fragments resulted in a 2476 bp fragment, which was digested with BglII and SalI. This insert PCR fragment was ligated into BglII and SalI-digested pDONR/Zeo-gN-Int124. The resultant plasmid named pDONR/Zeo-gN-Int1234-dAE was recombined with pART27-35S-GWB-DHA through LR reaction to obtain pART27-35S-gN-Int1234-dAE.

All of the plasmids constructed in this study were digested with appropriate restriction enzymes to verify the presence of the designed expression cassettes. Furthermore, coding sequences in the plasmids amplified by PCR were confirmed by sequencing with ABI3130 DNA sequencer (Thermo Fisher Scientific, Waltham, MA, USA). The nucleotide sequence of the expression cassette for *gN-Int1234* containing the CaMV 35S promoter and the octopine synthase terminator is included as a representative in Supplemental Fig. 3.

### Agroinfiltration

Transformation by electroporation and infiltration of *Agrobacterium tumefaciens* (syn. *Rhizobium radiobacter*) strain GV3101 (pMP90) were performed as described previously^7^. Agrobacteria transformed with each of the pART27 empty vector^40^, pART27-35S-TomH1 carrying the *p50* cDNA^7^, pART27-35S-cN (originally named as pART27-35S-N-DHA)^7^, other pART27-based binary plasmids (see above), and pGLW3-erGFP carrying the ToMV-erGFP sequence^15^ were cultured in YEP media (10 g/L Bacto Peptone, 10 g/L Bacto Yeast Extract, and 5 g/L NaCl) containing rifampicin (25–50 µg/mL) and kanamycin (25–50 µg/mL), and resuspended in incubation buffer (10 mM MgCl_2_, 10 mM MES, and 150 µM acetosyringone) to adjust OD_600_ to approximately 0.1. Agrobacteria carrying an *N* derivative were infiltrated to expanded leaves alone or in combination with Agrobacteria carrying the *p50* cDNA at ratio of 1:1 after final OD_600_ of inoculum suspension was adjusted to 0.1, except for a virus infection assay using pGLW3-erGFP. In the virus infection assay, agrobacteria carrying pGLW3-erGFP were mixed with those carrying an *N* derivative or *p50* transgene at a ratio of 1:50 to adjust final OD_600_ of the inoculum suspension to 0.102. Infiltrated plants were kept in a growth chamber with a 16 h light/8 h dark photoperiod. Cell death induction, RT-qPCR and virus infection assays were carried out at 25 °C.

### Total RNA extraction and quantification of gene expression

Extraction of total RNA and RT-qPCR analysis were performed as described previously^16^. A primer mix of oligo dT and random hexamers, included in an RT kit, was used for cDNA synthesis. Gene-specific oligonucleotides for PCR were as follows: Actin/real/F (5′-CTATTCTCCGCTTTGGACTTGGCA-3′) and Actin/real/R (5′-AGGACCTCAGGACAACGGAAACG-3′) to amplify a portion of for *actin* mRNA (GenBank accession number X69885); N/real/F1 (F1: 5′-TTCTTTGTACCTTTTGCTGGCTTAT-3′) and N/real/R1 (R1: 5′-CTCTGGTCCTTCTTTATACAACAAAC-3′) to amplify a 3′-region of exon 4 of *N* mRNA (GenBank accession number U15605); attB1 adaptor primer (F2: 5′-GGGGACAAGTTTGTACAAAAAAGCAGGCT-3′) and Nfs/real/R1 (R2: 5′-TTCCCTTATCATTCAAGACTTCG-3′) to amplify the sequence from a part of a vector-derived 5′-UTR to a 5′-region of exon 1 of *N* mRNA; N/E1-I1/real/F (F3: 5′-TCACATGTTCGGAACCAAAA-3′) and N/E1-I1/real/R (R3: 5′-CATTTGAATGCAAAGTATTCAGC-3′) to amplify the sequence from a 3′-region of exon 1 to a 5′-region of intron 1 of premature *N* mRNA; N/E2-I2/real/F (F4: 5′-TTCAAAAAGATCCCGGAGAA-3′) and N/E2-I2/real/R (R4: 5′-CTGGAATTGACTGCCCTATG-3′) to amplify the sequence from a 3′-region of exon 2 to a 5′-region of intron 2 of premature *N* mRNA.

Regarding transient expression assays of *gN-Int1234-dAE*, absence of alternative splicing within intron 3 was confirmed by RT-qPCR using Ntr/real/F1 (5′-GAACAATATTGCAGAGAAAGAGGG-3′) and Ntr/real/R1 (5′-TTAGACCAGCTGAGATCTATC-3′)^34^ (Supplemental Fig. 4).

The nucleotide sequences amplified by the above primer sets for RT-qPCR are shown in Supplemental Fig. 3.

### GFP imaging of virus infection sites

Leaf discs were excised from infiltrated areas of an *N. benthamiana* leaf. Fluorescent sites infected by ToMV-erGFP were monitored with an all-in-one fluorescence microscope (BZ-9000) (Keyence, Osaka, Japan). Total fluorescent areas or fluorescent spots, representing individual infection loci, were measured using the ImageJ version 10.2 software (http://rsb.info.nih.gov/ij/).

### Statistical analysis

Treatment values were log transformed and variances about treatment means tested for homogeneity using F-test (Figs. 2 and 3d) or Bartlett’s test (Figs. 3a–c, 4, 5, and 7) to allow analysis by parametric statistics^41^. Data were analyzed by Student’s *t*-test (Figs. 2 and 3d) or two-way ANOVA (block and treatment) (Figs. 3a–c, 4, 5, and 7) to determine if significant differences existed in the population of means, and if significant differences were observed in multiple comparison, then further analyzed using a Tukey–Kramer test to identify significant differences between individual treatment means using Microsoft Excel for Mac 2011 (Microsoft, Redmond, WA, USA) with the add-in Statcel 3 software (OMS Publishing, Saitama, Japan). For data in Fig. 4, F2 + R2 primer set, the transformed data did not have homogeneity of variance and therefore two-way ANOVA and Tukey–Kramer tests were not performed. Instead, the non-parametric Steel–Dwass one-way analysis of variance^42^ was conducted using the Statcel 3 software (OMS Publishing).

### Compliance statement

The authors comply with relevant institutional, national, and international guidelines and legislation.

## Supplementary Information

Supplementary Figure 1.

Supplementary Figure 2.

Supplementary Figure 3.

Supplementary Figure 4.
