# Supplementary material for: Cooperative roles of introns 1 and 2 of tobacco resistance gene N in enhanced N transcript expression and antiviral defense responses
Source: Sci Rep. 2021 Jul 29;11:15424. doi: 10.1038/s41598-021-94713-4 (PMC8322402; doi:10.1038/s41598-021-94713-4)
Supplement: Supplementary file 1 — Supplementary Figure 1. [file 41598_2021_94713_MOESM1_ESM.pdf]

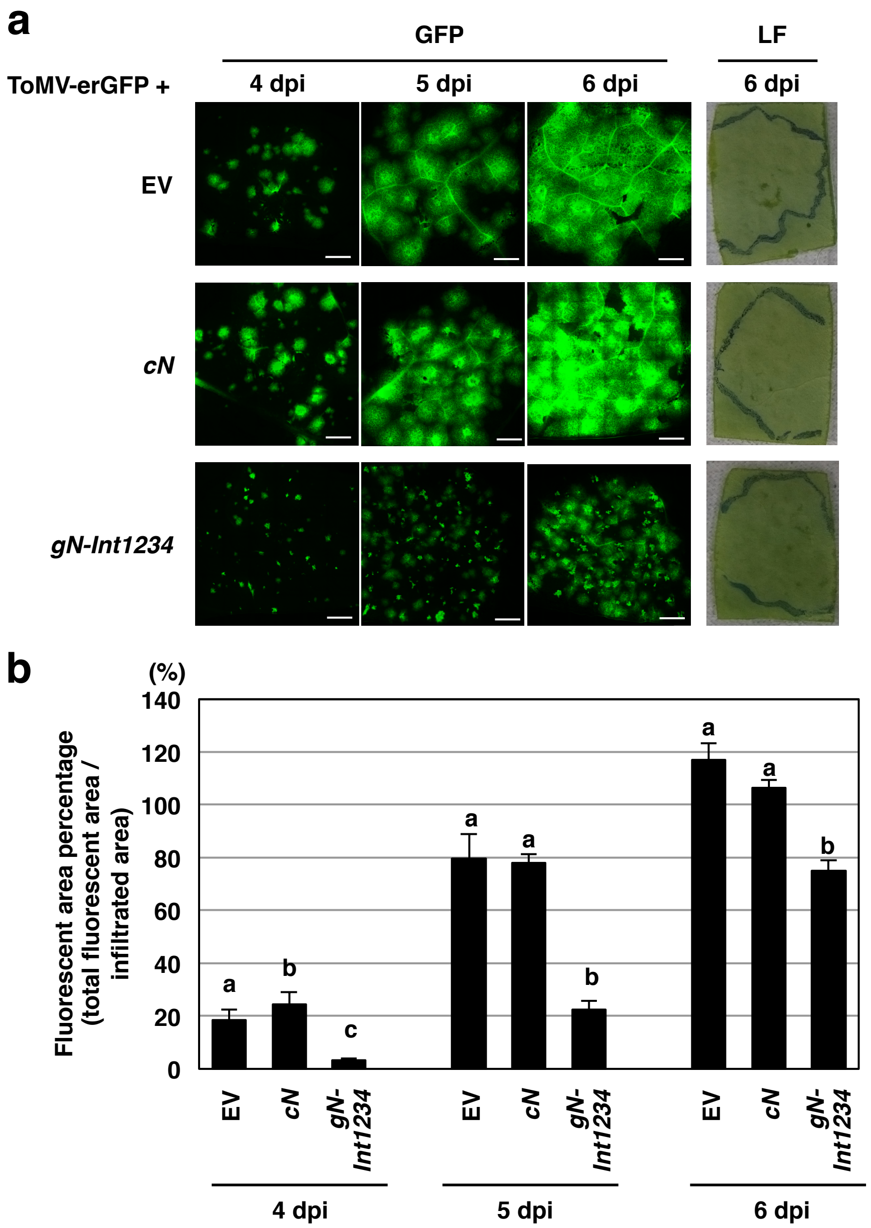

Supplemental Figure 1. Virus resistance against ToMV-erGFP in *Nicotiana benthamiana* transiently expressing *cN* or *gN-Int1234*

(a) Agrobacterium transformants carrying *cN* or *gN-Int1234* were infiltrated with those carrying the infectious ToMV-erGFP clone at a ratio of 50:1 to *N. benthamiana*. The final OD600 of the bacterial suspension for infiltration was adjusted to 0.102. Agrobacteria carrying the empty vector (EV) were used as a control. Representative fluorescent images taken at 4, 5, and 6 days postinfiltration (dpi) and light field (LF) images of epidermis at 6 dpi are shown. Bar = 2.5 mm. (b) Percentages of fluorescent areas were calculated by dividing total fluorescent area by infiltrated area. Data in panel b were analyzed by the two-way ANOVA test followed by the Tukey-Kramer test ($p < 0.05$). Different letters above the bars indicate that the differences in the means between values are statistically significant at each time point .
