# Supplementary material for: Cooperative roles of introns 1 and 2 of tobacco resistance gene N in enhanced N transcript expression and antiviral defense responses
Source: Sci Rep. 2021 Jul 29;11:15424. doi: 10.1038/s41598-021-94713-4 (PMC8322402; doi:10.1038/s41598-021-94713-4)
Supplement: Supplementary file 2 — Supplementary Figure 2. [file 41598_2021_94713_MOESM2_ESM.pdf]

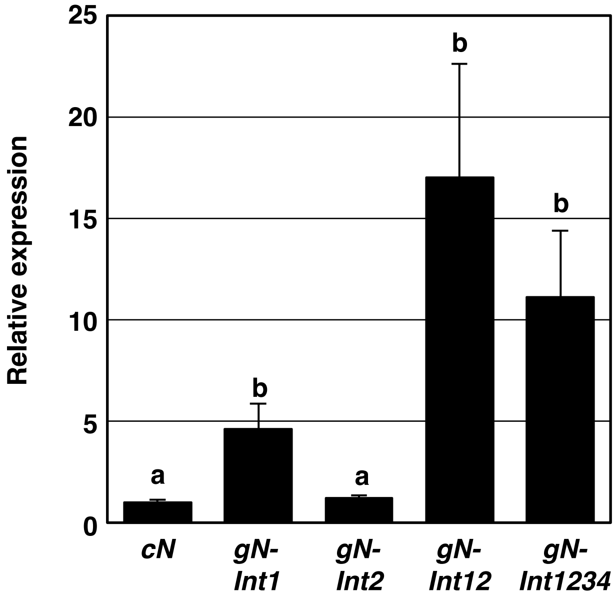

Supplemental Figure 2. Compilation analysis demonstrated statistically significant increases in transcript levels of *gN-Int1*, *gN-Int12* and *gN-Int1234* compared with those of *cN* and *gN-Int2*

All data from RT-qPCR experiments using F1 + R1 primer set for Fig. 3b, 4a and 4b, and 7d were used for this multiple comparison. The mean value of *cN* was used to normalize transcription levels of the other *N* transgenes (i.e., *gN-Int1*, *gN-int2*, *gN-Int12*, and *gN-Int1234*). As variances about treatment means were examined using Bartlett's test and found not homogeneous, data was analyzed using the Steel-Dwass non-parametric test to identify significant differences between individual treatment means for Microsoft Excel for Mac 2011. Different letters above the bars indicate that the means are statistically significantly different at the 0.05 level.
