# Supplementary material for: Cooperative roles of introns 1 and 2 of tobacco resistance gene N in enhanced N transcript expression and antiviral defense responses
Source: Sci Rep. 2021 Jul 29;11:15424. doi: 10.1038/s41598-021-94713-4 (PMC8322402; doi:10.1038/s41598-021-94713-4)
Supplement: Supplementary file 3 — Supplementary Figure 3. [file 41598_2021_94713_MOESM3_ESM.pdf]

5´-TCGACGAATTAATTCCAATCCCACAAAAATCTGAGCTTAACAGCACAGTTGCTCCT
CTCAGAGCAGAATCGGGTATTCAACACCCTCATATCAACTACTACGTTGTGTATAACGG
TCCACATGCCGGTATATACGATGACTGGGGTTGTACAAAGGCGGCAACAAACGGCGTTC
CCGGAGTTGCACACAAGAAATTTGCCACTATTACAGAGGCAAGAGCAGCAGCTGACGCG
TACACAACAAGTCAGCAAACAGACAGGTTGAACTTCATCCCCAAAGGAGAAGCTCAACT
CAAGCCCAAGAGCTTTGCTAAGGCCCTAACAAGCCCACCAAAGCAAAAAGCCCACTGGC
TCACGCTAGGAACCAAAAGGCCCAGCAGTGATCCAGCCCCAAAAGAGATCTCCTTTGCC
CCGGAGATTACAATGGACGATTTCCTCTATCTTTACGATCTAGGAAGGAAGTTCGAAGG
TGAAGGTGACGACACTATGTTCACCACTGATAATGAGAAGGTTAGCCTCTTCAATTTCA
GAAAGAATGCTGACCCACAGATGGTTAGAGAGGCCTACGCAGCAGGTCTCATCAAGACG
ATCTACCCGAGTAACAATCTCCAGGAGATCAAATACCTTCCCAAGAAGGTTAAAGATGC
AGTCAAaAAGATTCAGGACTAATTGCATCAAGAACACAGAGAAAGACATATTTCTCAAG
ATCAGAAGTACTATTCCAGTATGGACGATTCAAGGCTTGCTTCATAAACCAAGGCAAGT
AATAGAGATTGGAGTCTCTAAAAAGGTAGTTCCTACTGAATCTAAGGCCATGCATGGAG
TCTAAGATTCAAATCGAGGATCTAACAGAACTCGCCGTGAAGACTGGCGAACAGTTCAT
ACAGAGTCTTTTACGACTCAATGACAAGAAGAAAATCTTCGTCAACATGGTGGAGCACG
ACACTCTGGTCTACTCCAAAAATGTCAAAGATACAGTCTCAGAAGACCAAAGGGCTATT
GAGACTTTTCAACAAAGGATAATTTCGGGAAACCTCCTCGGATTCCATTGCCCAGCTAT
CTGTCACTTCATCGAAAGGACAGTAGAAAAGGAAGGTGGCTCCTACAAATGCCATCATT
GCGATAAAGGAAAGGCTATCATTCAAGATCTCTCTGCCGACAGTGGTCCCAAAGATGGA
CCCCCACCCACGAGGAGCATCGTGGAAAAAGAAGACGTTCCAACCACGTCTTCAAAGCA
AGTGGATTGATGTGACATCTCCACTGACGTAAGGGATGACGCACAATCCCACTATCCTT
CGCAAGACCCTTCCTCTATATAAGGAAGTTCATTTCATTTGGAGAGGACACGCTCGAGG
AATTCGGTACCCCATCAACAAGTTTGTACAAAAAAGCAGGCTACCATGGCATCTTCTT
CTTCTTCTTCTAGATGGAGCTATGATGTTTTCTTAAGTTTTAGAGGCGAAGATACTCGA
AAAACGTTTACAAGTCACTTATACGAAGTCTTGAATGATAAGGGAATAAAAACCTTTCA
AGATGATAAAAGGCTAGAGTACGGCGCAACCATCCCAGGTGAACTCTGTAAAGCTATAG
AAGAGTCTCAATTTGCCATTGTTGTTTTCTCAGAGAATTATGCAACATCAAGGTGGTGT
TTGAATGAACTAGTGAAGATCATGGAATGCAAAACTCGATTTAAGCAAACTGTTATACC
GATATTCTATGATGTGGATCCATCACATGTTCGGAACCAAAAGGAGAGCTTTGCAAAAG
CCTTTGAAGAACATGAAACAAAGTATAAGGATGATGTTGAGGGAATACAAAGATGGAGG
ATTGCTTTAAATGAAGCGGCCAATCTCAAAGGCTCATGTGATAATCGTGACAAgtgagt
taaaaacatataagctgaatactttgcattcaaatgagttaaacataatcttaaataaa
tttttcaatttttttggaataaattgatagttgattatatatgtttctatcagttaatta
caaactcaataacattattacgtagataaaatttttattagttcttcaaagagtttgat
ttatgtgcacactctttgtatatatcacaatctttttacttttgtagGACTGATGCAGA
CTGTATTCGACAGATTGTTGACCAAATCTCATCCAAATTATGCAAGATTTCTTTATCTT
ATTTGCAAAACATTGTTGGAATAGATACTCATTTAGAGAAAATAGAATCCTTACTAGAG
ATAGGAATCAATGGTGTTCGGATTATGGGGATCTGGGGAATGGGGGGAGTCGGTAAAAC
AACAATAGCAAGAGCTATATTTGATACTCTTTTAGGAAGAATGGATAGTTCCTATCAAT

TTGATGGTGCTTGTTTCCTTAAGGATATTAAAGAAAACAAACGTGGAATGCATTCTTTG
CAAAATGCCCTTCTCTCTGAACTTTTAAGGGAAAAAGCTAATTACAATAATGAGGAGGA
TGGAAAGCACCAAATGGCTAGTAGACTTCGTTCGAAGAAGGTCCTAATTGTGCTTGATG
ATATAGATAATAAAGATCATTATTTGGAGTATTTAGCAGGTGATCTTGATTGGTTTGGT
AATGGTAGTAGAATTATTATAACAACTAGAGACAAGCATTTGATAGAGAAGAATGATAT
AATATATGAGGTGACTGCACTACCCGATCATGAATCCATTCAATTGTTCAAACAACATG
CTTTCGGAAAAGAAGTTCCAAATGAGAATTTTGAGAAGCTTTCATTAGAGGTAGTAAAT
TATGCTAAAGGCCTTCCTTTAGCCCTCAAAGTGTGGGGTTCTTTGCTGCATAACCTACG
ATTAACTGAATGGAAAAGTGCTATAGAGCACATGAAAAATAACTCTTATTCTGGAATTA
TTGATAAGCTCAAAATAAGTTATGATGGATTAGAGCCCAAACAACAAGAGATGTTTTTA
GATATAGCATGCTTCTTGCGAGGGGAAGAAAAAGATTACATCCTACAAATCCTTGAGAG
TTGTCATATTGGAGCTGAATACGGGTTACGTATTTTAATTGACAAATCTCTTGTGTTCA
TCTCTGAATATAATCAGGTTCAAATGCATGACTTAATACAGGATATGGGTAAATATATA
GTGAATTTTCAAAAAGATCCCGGAGAACGTAGCAGATTATGGCTCGCCAAGGAAGTCGA
AGAAGTGATGAGCAACAACACAgtaagtaagctaaataatgcaataatatttaatttct
aattttatattctaaagacacatagggcagtcaattccagttatttgttcctcttgctt
catagtcttgcaggtacatcattttagttgtttactttagttagtaggagatataaaag
taatattaattacctcattagtaaaaaaaaacattaattgcctaatttgtttagtagcc
gctttaatttacgttccctaattcgtttttcttatatttttagggatggattagtct
agtagccacttaatctgtttgatccaatgtctttctttggattaacttgaaaattttat
gacattatatataataactcaatcattcattcactttaccattattatttttatataa
agttacaatttattggtactgtttcagttacaattactttccaacatggaaaacttata
aactggactccaataaacttctaagaaaaatgtaataatagaaaataaaattatataat
taattacaaaaaagtatttttctgaagtaacatcagtatttcttaaaaagaatccaatt
aacattgtatcttaaactttggtattgtaaggagtgagaaagtagtggccttatttcaa
tttgacgtgaagaatagaatgccttttaacgacataagggaaggggcaagaataagtt
tctattcagccgggctcgaagcagaaggtagaacgtaatatcttttgttggttcagctc
atcaagctattacaaaagagtccgctcatattaacaaacggagtttatacgacatttga
aattatactttgtagactaatgatcttcttgttaccaggGGACCATGGCAATGGAAGCA
ATTTGGGTTTCTTCTTATTCTAGTACTCTACGCTTTAGCAATCAGGCCGTGAAAAATAT
GAAAAGGCTTAGGGTATTTAACATGGGGAGGTCGTCGACACATTATGCCATCGATTATC
TGCCCAACAACTTGCGTTGTTTTGTTTGCACTAACTATCCTTGGGAGTCATTTCCATCT
ACATTTGAACTCAAAATGCTTGTTCACCTCCAACTCCGACACAATTCTCTGCGTCATTT
ATGGACAGAAACAAAGgtacaatagcttgaattctattttgttgtcatttatttttctc
tctaactatctttgtcctttaatttggtgataatgaacaaatattattgtttttgtta
tgaaacaataaaagaagaagaacaatattgcagagaaagagggagatggaattcttatt
gaattttggggcgatttacaatggggtaagacccctctatttacaggggaaaaataact
tagcctcaaaataaagctctttaaaagatagacattcactctaaatagaattctattat
aacacttttggcgtacttcctttttttggctagaattatgatacatgtctttaaatgaac
agaagttgcttttgtaatttatcaggacttatgttgaaacttatgaaaattgttattgt

ttatgttgtctaatactaaatataaaatacaataatattttatcgtaatttttaaaaa
tttgtcaaataatgcaaatgaaaaattaaatttttttggtcctttaaaaatttgagaatg
aaaaagtacgagttatacttcctaaaagtttgatagtgaataatatgtaaaatttaaag
aatgactaatattggactaatactttaaaacaaataacttaatatacaaattatagcga
gacattttcattcgttgtactgaatgcaagaaagaaaggaaaaaaaaactcatttataa
tatagtttgtcttctactattttaccttattgcttcaaatttgtattttatcgattttg
ctatatcttatgattttttttcacggtcaatattcttcttacaagaataaattttatata
cctcaagtgttttgtcaatttgataaataatttttcttatatgatgaacttgtaaaata
atagaattggattcttttgctaattagttaattcaacgacttaattatttattctcaac
attaaaggaaataatttagtttttattaattcaaactcttagtatttgctcattctaat
tttcagtccaataagaattcaattttcaaatagtaagaaaagtcatatattttgaattt
tatgttttccgaagcattgtttgtttgtttaactctacgggagttttctaactcacatt
ttgtataataaaatttttttgagtagtagttcagtacaactctaatattaatgggcttta
aataaggaaatatatattacgtaaaaatttaaatcattttaaagttctttcctaccaag
taaataagggaaaatttaataacaaaaatttagttgattttaaaatcctaaatattaga
aaattaacttaaaatataatttcgtctagtgtaaaatttatttttaaagggtaaaaaag
acgaacgacattaagagcctttgtaattttaatatagtataaatataaataatttacct
ttattcagtttcttaacaagtaattttccatatataaaaaataaatttctatattcaca
caaaaataatgtgttggccctcgtaattcaaatactatcattcatttcttgtcgaggga
gtagtaaatactttttaggaaagttagcaataagtaatcaagaaatcaagaaaacagagg
tcatttgatgcccacaaatacaaatgaaaaaacaaaacaaatgttacgaaacaataaaa
gaacaagaatagcctcaaagtaaaactctctgatagacatttactctaaatagaattct
atttataacaatcaaaaagtttctacatttatagatagctccactagccaaatatttta
ttattggaatcagcaaaataggttgtttcttttttttattctcattctgtctgtgttcta
aacagCATTTGCCGTCTCTACGGAGGATAGATCTCAGCTGGTCTAAAAGATTGACGCGA
ACACCAGATTTCACGGGGATGCCAAATTTGGAGTATGTGAATTTGTATCAATGTAGTAA
TCTTGAAGAAGTTCACCATTCCCTGGGATGTTGCAGCAAAGTCATTGGTTTATATTTGA
ATGATTGTAAAAGCCTTAAGAGGTTTCCATGTGTTAACGTGGAATCTCTTGAATATCTG
GGTCTAAGAAGTTGCGATAGTTTAGAGAAATTGCCAGAAATCTACGGGAGAATGAAGCC
GGAGATACAGATTCACATGCAAGGCTCTGGGATAAGGGAACTACCATCATCTATTTTTC
AGTACAAAACTCATGTTACCAAGCTATTGTTGTGGAATATGAAAAACCTTGTAGCTCTT
CCAAGCAGCATATGTAGGTTGAAAAGTTTGGTTAGTCTGAGTGTGTCGGGTTGCTCAAA
ACTTGAAAGCTTGCCAGAAGAGATAGGGGATTTAGACAACTTACGGGTGTTTGATGCCA
GTGATACTCTAATTTTACGACCTCCGTCTTCCATCATACGCTTGAACAAACTTATAATC
TTGATGTTTCGAGGCTTCAAAGATGGAGTGCACTTTGAGTTCCCTCCTGTGGCTGAAGG
ATTACACTCATTGGAATATCTGAATCTCAGTTACTGCAATCTAATAGATGGAGGACTTC
CGGAAGAGATTGGATCCTTATCCTCTTTGAAAAAGTTGGATCTCAGTAGAAATAATTTT
GAGCATTTGCCTTCAAGTATAGCCCAACTTGGTGCTCTTCAATCCTTAGACTTAAAAGA
TTGCCAGAGGCTTACACAGCTACCAGAACTTCCCCCAGAATTAAATGAATTGCATGTAG
ATTGTCATATGGCTCTGAAATTTATCCATTATTTAGTAACAAAGAGAAAGAAACTACAT

AGAGTGAAACTTGATGATGCACACAATGATACTATGTACAATTTGTTTGCATATACCAT
GTTTCAGAATATCTCTTCCATGAGGCATGACATCTCTGCTTCAGATTCCTTGTCACTAA
CAGTATTTACCGGTCAACCGTATCCTGAAAAGATCCCGAGTTGGTTCCACCATCAGGGT
TGGGATAGTAGTGTATCAGTCAATTTGCCTGAAAATTGGTATATACCTGATAAATTCTT
GGGATTTGCTGTATGTTACTCTCGTAGCTTAATTGACACAACAGCTCACTTGATTCCCG
TATGTGATGACAAGATGTCGCGCATGACCCAGAAACTTGCCTTATCAGAATGTGATACA
GAATCATCCAACTATTCAGAATGGGATATACATTTTTTCTTTGTACCTTTTGCTGGCTT
ATGGGATACATCTAAGGCAAATGGAAAAACACCAAATGATTATGGGATTATTAGGCTAT
CTTTTTCTGGAGAAGAGAAGATGTATGGACTTCGTTTGTTGTATAAAGAAGGACCAGAG
GTTAATGCCTTGTTACAAATGAGGGAAAATAGCAATGAACCAACAGAACATTCCACTGG
GATAAGGAGGACTCAATATAACAACAGAACTTCCTTTTATgtaagtctctacttctatt
agctacaaagtcttcttccaaaatcaatactccatccgttccagtttatgtgaacctat
tttttgttcgtccattctaaaaagaatgacccctttctaaatttggaaataattttggt
taaacttataattctaccattaacgagaagcttttataaccacacaaatattctggggc
ccttttttgaattgtttaggaccataaattccaaaagtcctcatttttttcttaaactccg
tgcccaatcaaacaagttcacgtaaattggaacggagggaatatatttttttcttctcat
tcttttcccctatttacagGAGCTCATCAATGGGAACCCAGCTTTCTTGTACAAAGTGG
T*TGATGGGTTCGAAATCGATAAGCTTGGAGGTACTGGAGGATCCTACCCATACGACGTT*
*CCAGACTACGCTGGTTACCCATACGACGTTCCAGACTACGCTTGATCTAGAGTC***CTGC**
TTTAATGAGATATGCGAGACGCCTATGATCGCATGATATTTGCTTTCAATTCTGTTGTG
CACGTTGTAAAAAACCTGAGCATGTGTAGCTCAGATCCTTACCGCCGGTTTCGGTTCAT
TCTAATGAATATATCACCCGTTACTATCGTATTTTTATGAATAATATTCTCCGTTCAAT
TTACTGATTGTACCCTACTACTTATATGTACAATATTAAAATGAAAACAATATATTGTG
CTGAATAGGTTTATAGCGACATCTATGATAGAGCGCCACAATAACAAACAATTGCGTTT
TATTATTACAAATCCAATTTTAAAAAAAGCGGCAGAACCGGTCAAACCTAAAAGACTGA
TTACATAAATCTTATTCAAATTTCAAAAGtGCCCCAGGGGCTAGTATCTACGACACACC
GAGCGGCGAACTAATAACGcTCACTGAAGGGAACTCCGGTTCCCCGCCGGCGCGCATGG
GTGAGATTCCTTGAAGTTGAGTATTGGCCGTCCGCTCTACCGAAAGTTACGGGCACCAT
TCAACCCGGTCCAGCACGGCGGCCGGGTAACCGACTTGCTGCCCCGAGAATTATGCAGC
ATTTTTTTGGTGTATGTGGGCCCCAAATGAAGTGCAGGTCAAACCTTGACAGTGACGAC
AAATCGTTGGGCGGGTCCAGGGCGAATTTTGCGACAACATGTCGAGGCTCAGCAGGACC
TGCAGGCATGCAAG-3′

Supplemental Figure 3. The nucleotide sequence of the expression cassette for *gN-Int1234* containing the cauliflower mosaic virus 35S promoter (shaded) and the octopine synthase terminator (bold). The nucleotide sequences in red, orange, green, and blue indicate those amplified by RT-qPCR with the primer sets, N/real/F1 and N/real/R1, attB1 adaptor primer and Nfs/real/R1, N/E1-I1/real/F and N/E1-I1/real/R, and N/E2-I2/real/F and N/E2-I2/real/R, respectively. The separated underlined nucleotide sequences in purple represent the sequences that are linked together in the

alternatively spliced transcript and thus can be amplified by RT-qPCR with Ntr/real/F1 and Ntr/real/R1. Expected sizes of PCR products amplified with N/real/F1 and N/real/R1, attB1 adaptor primer and Nfs/real/R1, N/E1-I1/real/F and N/E1-I1/real/R, Ntr/real/F1 and Ntr/real/R1, and N/E2-I2/real/F and N/E2-I2/real/R are 150, 191, 151, 106, and 141 bp, respectively. Sequences represented by lower case letters are introns. The boxed ATG and TGA represent the start and termination codon, respectively. The 3´-terminal sequence downstream of exon 5 in the *N* sequence, which contains the *att*B2 recombination site and a double hemagglutinin coding sequence, is in italic.
