# Supplementary material for: Cooperative roles of introns 1 and 2 of tobacco resistance gene N in enhanced N transcript expression and antiviral defense responses
Source: Sci Rep. 2021 Jul 29;11:15424. doi: 10.1038/s41598-021-94713-4 (PMC8322402; doi:10.1038/s41598-021-94713-4)
Supplement: Supplementary file 4 — Supplementary Figure 4. [file 41598_2021_94713_MOESM4_ESM.pdf]

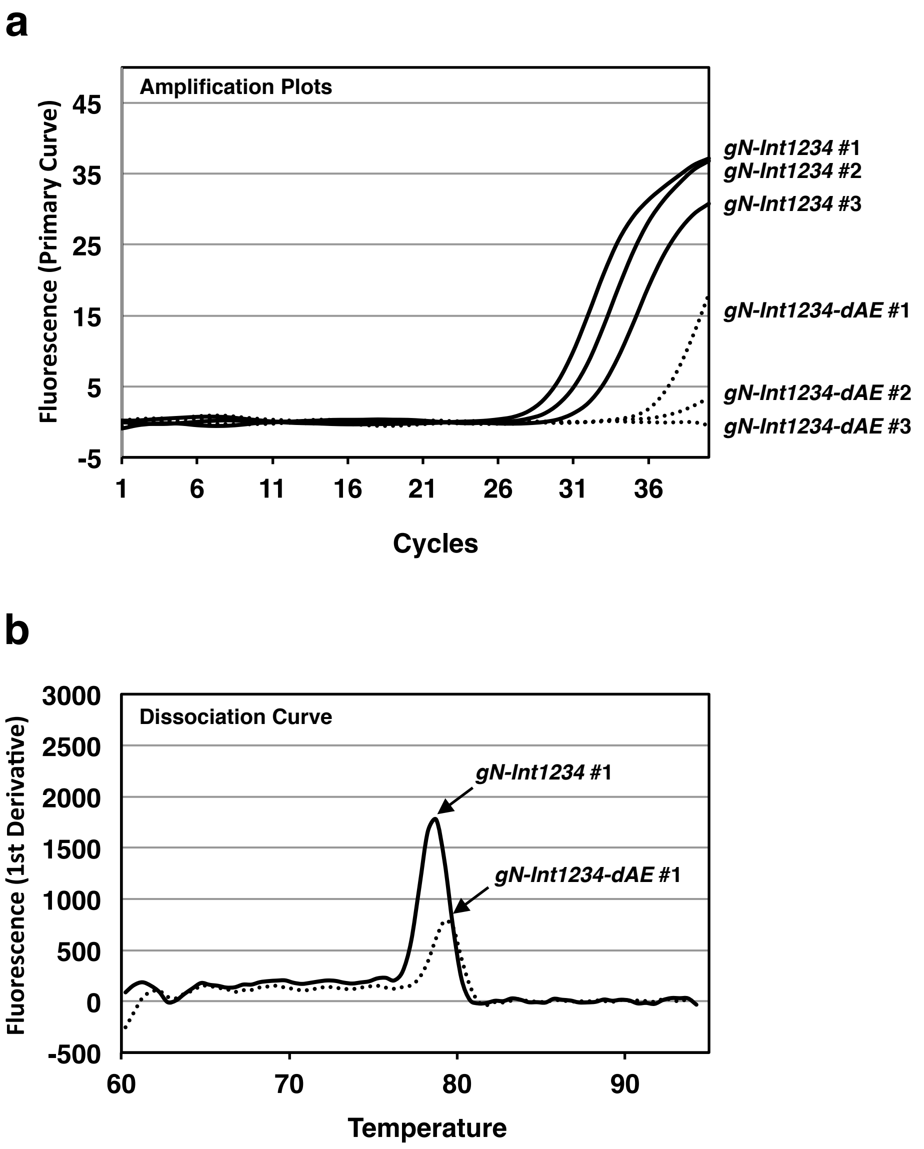

Supplemental Figure 4. Absence of alternatively spliced transcripts from *gN-Int1234-dAE* Agrobacterium transformants carrying a plasmid for *gN-Int1234* or *gN-Int1234-dAE* were infiltrated into nn tobacco leaves. The final OD600 of each bacterial suspension for infiltration was adjusted to 0.1. The alternatively spliced *N* transcripts at 36 h post-infiltration were quantified by RT-qPCR with Ntr/real/F1 + Ntr/real/R1 primer set. (a) Solid and dotted lines indicate primary amplification plot curves of fluorescence for *gN-Int1234* and *gN-Int1234-dAE*, respectively. Three samples for each transgene, indicated as #1, #2, and #3, were examined. (b) For *gN-Int1234-dAE* #1 that exhibited a slight increase in fluorescence at very late cycles, melting curve analysis was carried out to determine that the melting temperature of PCR products from *gN-Int1234-dAE* #1 was different from that from *gN-Int1234 #1*. This difference in melting temperature indicates that product for *gN-1234-dAE* #1 did not represent the sequence of alternatively spliced transcripts.
